# Challenges posed by COVID -19 to patients with cancer: lessons from a Moroccan experience

**DOI:** 10.11604/pamj.supp.2020.35.2.22585

**Published:** 2020-06-04

**Authors:** Wafaa Kaikani, Sqalli Houssani Mohammed

**Affiliations:** 1 Wafaa Kaikani, Université Mohammed VI des sciences de la santé, Casablanca, Morocco

**Keywords:** Cancer, care, COVID-19, crisis management

## Abstract

Outbreaks of infectious etiology, particularly those caused by a novel virus that has no known treatment or vaccine may result in the interruption of medical care and the life-threatening event among patients with cancer. Oncologists in low- and middle-income countries (LMICs) are worried about how COVID-19 is expected to disproportionately affect cancer patients, how can they best care for cancer patients in an uncertain and dangerous healthcare environment. This article discusses some strategies that oncologists from low- and middle-income countries can take to keep cancer patients and staff safe while continuing to provide compassionate, high-quality care under circumstances we’ve never had to face before. The approach is taken toward managing this high-risk situation could be easily adopted by health care organizations. We hope that, with those simple steps, we will continue to provide compassionate, high-quality care under circumstances we’ve never had to face before.

## Perspective

The coronavirus disease 2019 (COVID-19) pandemic has rapidly escalated into a global crisis. This outbreak of COVID-19 in different parts of the world pose a major challenge to health care systems and expose patients and staff to serious risk. Especially in Low- and Middle-Income Country; where health systems are already stretched thin, a pathogen like the coronavirus can quickly easily overwhelm them [1]. These outbreaks pose a greater threat to patients with cancer; there are still many unknowns in terms of how COVID-19 affects people with cancer. Unsettling data from Wuhan show that patients with cancer are at higher risk of developing disease-specific complications [2,3], Many cancer patients also have additional complications, the combination of immunosuppression, other health problems and age make COVID-19 a major concern for many of them. The provision of cancer care is complex and requires multiple teams of professionals and access to sophisticated, expensive resources. The cancer care already presents major challenges to the health care systems in affluent countries. Many challenges will arise in developing countries while facing Covid -19 outbreak, which present an additional burden to inherently complex care [4,5]. The factors related to oncology patients´ care and corresponding outcomes are a major concern for the oncology community; therefore, this article describes the approach used to manage oncology services affected areas in developing countries. In coordination with the organizational leadership, we developed a plan to manage the crisis with the main objective to keep our cancer patients and staff safe while continuing to provide compassionate, high-quality care under circumstances we’ve never had to face before.

### Staff management

*Protection of health providers:* the aim of the staff management plan was to protect health and frontline staff and assure a safe work environment in order to prevent provider burnout. Minimize staff exposure to infection, and educate them about the crisis These measures included: i) Enforcing a strict “stay at home when ill” policy and insuring staff have access to get testing; ii) Streamline their work process by clarifying their roles and responsibilities; iii) Verifying their knowledge of the transmission mechanism and protective measures to ensure that clinical practice was based on evidence and not myth; iv) Making sure that all the staff attend the hospital wide educational training on donning and doffing clothing, applying strict hand hygiene, and implementing infection prevention precaution; v) All staff were instructed and educated about proper hand hygiene: Prioritizing the use of soap and water over hand gel; vi) Minimizing their anxiety and stress; vii) Limiting the number of team members who enter patients room; viii) Adopting a no visitor policy with rare exceptions such as end-of-life circumstances.

### Patient management

*The patient management plan had 3 main aims:* to prevent new infection in the oncology service, to manage currently infected patients, and to provide timely; cancer care for the whole patient population.

*Appointments scheduling:* all the appointments were reviewed, Medical records of all scheduled patients were reviewed by an oncologist, and patients were divided into 3 categories. i) We kept the scheduled appointments for patients who were scheduled for chemotherapy that could not be postponed or were due for disease assessment; ii) We postponed appointments for patients who could be rescheduled after a short delay; iii) Some appointments were rescheduled as far in the future as deemed clinically safe for the patient without any negative impact for routine follow-up to protect patient who require hospital visits. All patients were screened before entering the clinic with a checklist that included clinical and epidemiologic criteria; anyone with a suspected infection was referred to a triage area segregated from the general population.

*Communication within the patient:* the impact of the lockdowns on access to cancer treatments could be a major cause for concern. Patients must be in constant and close communication with their care teams, nurses and oncologists if they are concerned about potential exposure to the virus as well as to learn what care is essential and what may be safely postponed. Internet access is a good way to stay in contact with the cancer care team. One particularly useful application is WhatsApp. What Sapp Messenger or simply WhatsApp is a freeware, cross-platform messaging and Voice over IP (VoIP) service. It allows users to send text messages and voice messages, make voice and video calls, and share images, documents, user locations, and other media. Health care services have to develop disease-specific telephone questions that doctors can ask all cancer patients to determine whether a visit is absolutely necessary and whether it can be postponed. It´s important for patients to know whom to call to reach the cancer care. A virtual monitoring program can increase access and decrease travel.

*Patient education:* the need to pursue strict measures of self-hygiene and avoid any crowding in order “ flatten the curve” and reduce the pace of virus transmission is of utmost importance [6]. Therefore, where possible patient information via handouts, signing sheets should be replaced with web-based communication if possible. i) Patients are encouraged to limiting the number of caregivers who can accompany them to treatment sessions; ii) Compiling an extensive list of frequently asked questions that offer advice and support to patients.

**Infection control management:** all patients coming to the outpatient clinic must be screened; and if any patient met the definition for suspected infection, she or he was placed on airborne isolation precautions and We appeal to clinical oncologists to learn the diagnosis of COVID-19 well. Cancer patients should be adequately screened for their epidemiological history, especially for recent travel to areas with medical records, and respiratory and constitutional symptoms. Fever patients during chemotherapy need to receive differential diagnosis and screening according to national standards.

### Cancer management

*Individual patient decision:* first, decisions about how to proceed is the responsibility of clinicians in multidisciplinary teams including respiratory specialists, and to be treated with other therapeutic options. Clinicians will have to balance the relative risks of developing COVID-19 disease while severely immunosuppressed, or of developing a severe treatment complication, against the risks of tumor progression. Cancer care coordination should use virtual technologies as much as possible, and facilities with tumor boards may find it helpful to locate multidisciplinary experts by virtual means, to assist with decision making and establishing triage criteria. Many triage guidelines and joint recommendations are being issued as we appear to be entering a new phase of the COVID-19 pandemic, we advise to apply them taking into account the prevailing state of the healthcare service; for instance, we modified our chemotherapy programs where appropriate. For metastatic cancer patients, maintenance therapy is the optimal choice. The patients with tumor progression or poor biological behavior should receive or continue combination chemotherapy. Adjuvant chemotherapy should reduce the intensity of treatment and shorten the therapy time. Patients with stable diseases and good general conditions may delay imaging examination. We are also using growth factor support to prevent neutropenia as much as possible in patients. We encourage using local services for blood tests if possible. Results could be sent using WhatsApp application. The coming months will pose many further challenges, which might include accessibility to scarce resources, effects on drug manufacture and supply, and the effect on care of patients with cancer from low in come and middle-income countries. Continued collaboration among the international oncology community is required to get through such uncertain times

## Competing interests

The author declares no competing interests.
